# Polymeric Mesoporous Silica Nanoparticles for Enhanced Delivery of 5-Fluorouracil In Vitro

**DOI:** 10.3390/pharmaceutics11060288

**Published:** 2019-06-19

**Authors:** Thashini Moodley, Moganavelli Singh

**Affiliations:** Nano-Gene and Drug Delivery Group, Discipline of Biochemistry, School of Life Sciences, University of KwaZulu-Natal, Private Bag X54001, Durban 4000, KwaZulu-Natal, South Africa; thashinim@gmail.com

**Keywords:** 5-fluorouracil, mesoporous nanoparticles, cancer, chitosan, poly(ethylene)glycol

## Abstract

There is a need for the improvement of conventional cancer treatment strategies by incorporation of targeted and non-invasive procedures aimed to reduce side-effects, drug resistance, and recurrent metastases. The anti-cancer drug, 5-fluorouracil (5-FU), is linked to a variety of induced-systemic toxicities due to its lack of specificity and potent administration regimens, necessitating the development of delivery vehicles that can enhance its therapeutic potential, while minimizing associated side-effects. Polymeric mesoporous silica nanoparticles (MSNs) have gained popularity as delivery vehicles due to their high loading capacities, biocompatibility, and good pharmacokinetics. MSNs produced in this study were functionalized with the biocompatible polymers, chitosan, and poly(ethylene)glycol to produce monodisperse NPs of 36–65 nm, with a large surface area of 710.36 m^2^/g, large pore volume, diameter spanning 9.8 nm, and a favorable zeta potential allowing for stability and enhanced uptake of 5-FU. Significant drug loading (0.15–0.18 mg_5FU_/mg_msn_), controlled release profiles (15–65%) over 72 hours, and cell specific cytotoxicity in cancer cells (Caco-2, MCF-7, and HeLa) with reduced cell viability (≥50%) over the non-cancer (HEK293) cells were established. Overall, these 5FU-MSN formulations have been shown to be safe and effective delivery systems in vitro, with potential for in vivo applications.

## 1. Introduction

5-Fluorouracil (5-FU) is a potent anti-metabolite that was first patented in 1956 for application in chemotherapy regimens [1]. It is administered as a pro-drug and is rapidly converted to its intermediates 5-fluoro-2′-deoxyuridine-5′monophosphate and 5-fluorouridine triphosphate, with the former actively inhibiting thymidylate synthase and ultimately DNA replication and repair. The latter has been linked to RNA, inhibiting replication and cellular functioning accordingly [2,3,4]. The ultimate bioavailability and induced-toxicity have been linked to the unique properties of 5-FU. 5-FU is a low molecular weight, negatively charged (130.08 g/mol; pKa ~8) compound which is quickly excreted from the body (7–20% unchanged in urine within six hours, up to 90% excreted within the first hour), with only a small percentage of the administered dose being metabolized, primarily in the liver. It has low lipid solubility and has a dose-dependent half-life average of between 10–20 minutes [1,2,5]. The associated intravenous toxicities noted with 5-FU include neuropathy, depression of white blood cells, cardiac toxicity, and hepatic or renal associated toxicity [6,7,8]. 

Alternative drug delivery strategies which reduce the administered dose and dosing interval, and which can passively or actively target tumor tissue, are attractive, and can be accomplished with the use of nanoparticle (NP)-based drug delivery systems [9]. These systems aim to provide targeting potentials, sustained drug release profiles, and biostability, and can administer a comparatively higher intra-tumor drug concentration with reduced drug-induced toxicity and side-effects commonly seen in vivo [10,11]. 

The use of mesoporous silica nanoparticles (MSNs) in drug delivery has become increasingly desirable due to the large surface area [12] that can be selectively modified to ensure higher loading of cargo into the mesoporous framework for sustained release profiles in disease models [13,14]. Thus, in this chapter a polymeric coated MSN with a net positive charge was synthesized to favorably load the chemotropic drug, 5-FU. Polymers, chitosan (CHIT), and polyethylene glycol (PEG), which at defined concentrations confer biodegradability [15], bioavailability [16,17], biocompatibility [18,19], hemocompatibility [20,21], increased circulating half-lives [22], and improved cellular uptake rates in tumor tissue [23,24], were incorporated into the delivery system. The use of the popularised non-ionic polymer PEG in conjunction with the cationic biopolymer CHIT as coating agents in drug delivery systems confers beneficial physico-chemical properties such as reduced interparticle attraction and provision of a hydrophilic protective corona that in turn improves the loading and release efficiency of the encapsulated drug, as well as improving the drug delivery systems’ (DDS) pharmacokinetic fate, longevity in serum, serum stability, and solubility of the encapsulated drug [1,2,3]. The use of multi-layered polyelectrolyte coating (PEC) for DDS imparts advantageous particle surface properties and has been employed in both hydrogel and microsphere formulations [1,2,3,4,5]. 

In this work, the release profile of 5-FU from MSN was investigated in vitro, with the release kinetics from the PEC polymeric-coated MSN matrix assessed for their potential biological performance. These MSNs were assessed for cytotoxicity and induced cell-death mechanisms in human cancer cell lines, with the overall therapeutic efficiency of 5FU-MSN formulations being evaluated to ascertain the suitability of MSN as a safe and efficient drug delivery vehicle and an appropriate alternative to conventional free drug administration formulations. 

## 2. Materials and Methods

### 2.1. Materials

Tetraethyl orthosilicate (TEOS, Si(OCH_2_CH_3_)_4_), Triton X-100 (TX100), cetyl trimethylammonium bromide (CTAB, 99%), polyethylene glycol_2000_ (PEG_2000_), chitosan (75–85% deacetylated), sodium tripolyphosphate (TPP), Tweens 20, ammonia solution (28–30%), sulfuric acid, sodium carbonate (Na_2_CO_3_), 5-fluorouracil (5-FU), and deuterium oxide were all purchased from Sigma Aldrich (St Louis, MO, USA). Eagle’s minimum essential medium (EMEM), fetal bovine serum (FBS), penicillin/streptomycin solution (10,000 U/mL), and trypsin−ethylene diamine tetraacetic acid (EDTA) (0.25% trypsin, 0.1% EDTA) were obtained from Lonza (Viviers, Belgium). Phosphate-buffered saline (PBS) tablets were purchased from Calbiochem, San Diego, CA, USA. The MTT salt (3-(4,5-dimethylthiazol-2-yl)-2,5-diphenyltetrazolium bromide) and trichloroacetic acid (TCA) were purchased from Merck, Darmstadt, Germany. The HeLa, Caco-2, and MCF-7 cells were originally purchased from the ATCC (Manassas, VA, USA), while the HEK293 cells were donated by the Anti-Viral Gene Therapy Unit, University of the Witwatersrand, Johannesburg, South Africa. All sterile plasticware for tissue cultures were obtained from Corning Inc., Corning, NY, USA. All other reagents were of analytical grade.

### 2.2. Synthesis of Mesoporous Silica Nanoparticles (MSNs)

Mesoporous silica nanoparticles (MSNs) were synthesized based on a sol-gel reaction adapted from the literature [25,26]. Briefly, cetyl trimethylammonium bromide (CTAB, 100 mg) was dissolved in 48 mL 18 MΩ waterand 350 µL of 2 M NaOH, and vigorously stirred in a round-bottom flask at 80 °C. Thereafter, 500 µL tetraethyl orthosilicate (TEOS) was added, and the solution incubated for 2 h. The nanoparticles were collected by centrifugation (4000 rpm, 30 min) and washed three times with ethanol, followed by deionized water. The CTAB surfactant was removed by overnight reflux in acidic methanol (20 mL methanol, 1 mL 37% hydrochloric acid) at 80 °C. The particles were then collected by centrifugation, dried, and calcinated at 70 °C for 24 h to remove any template reagents.

### 2.3. Chitosan Functionalisation

Approximately 40 mL of acetic acid (10% *v*/*v*) was added to 200 mg of dry, powdered, MSNs and 15 mg of CHIT [27,28], and stirred at ambient temperature for 24 h. The CHIT-MSNs were then recovered from the solution by centrifugation and washed with ethanol and deionized water three times. The final CHIT-MSN product was dried at 60 °C for 24 h.

### 2.4. Functionalisation with 2% and 5% PEG

Dilute acetic acid (30 mL, 2% *v*/*v*; pH 4.6) was added to separate beakers containing 22.5 mg of CHIT and 179 mg or 449 mg of PEG2000, respectively, followed by the addition of 7.725 mg of TPP in 15 mL of deionized water. To this was added 300 mg of MSN, and the mixtures were stirred at room temperature for 24 h. The final products (PEG_CHIT_MSN) were collected by centrifugation (1000 rpm, 30 min), and then washed and dried at 60 °C for 24 h.

### 2.5. Formation of 5-Fluorouracil:MSN Nanoconjugates

Approximately 100 mg of the above functionalized MSNs (f-MSNs) were soaked in a 3 mg/mL (30 mL) saturated 5-FU solution for 30 h, with stirring to allow the drug to enter the mesopores and the MSN framework. At zero and 30 h, 1 mL of drug solution was extracted and centrifuged, and the supernatant analyzed by UV spectrophotometry at a wavelength of 266 nm, while the precipitate was returned to the drug solution. The final 5-FU-loaded MSNs were collected by centrifugation and dried (60 °C, 24 h). The drug loading capacity was calculated as a percentage and as mg drug/mg f-MSN) using the following equation:

(1) $$\mathrm{Loading}\;\mathrm{capacity}\;=\frac{\mathrm{Mass}\;\mathrm{of}\;\mathrm{drugs}\;\mathrm{in}\;f-\mathrm{MSNs}}{\mathrm{Initial}\;\mathrm{Mass}\;\mathrm{of}\;\mathrm{MSNs}}$$

### 2.6. 5-FU Release

Approximately 5 mg of the 5-FU:MSN nanoconjugates were dispersed in 25 mL of PBS at pH 4.2 and 7.4, respectively, with stirring at 37 °C for 72 h [29]. MSN suspensions (0.5 mL) were regularly removed, centrifuged, and analyzed by UV spectrophotometry (266 nm), with addition of 0.5 mL of fresh PBS into the drug solution to maintain the volume. The experiments were conducted in triplicate and the mean results reported. The drug release was calculated using the equation

(2) $$\%\;R_{t}=\frac{C_{t}\cdot V_{1}+V_{2}\cdot \left(C_{t-1}+C_{t-2}+\cdots +C_{0}\right)}{W_{0}\cdot L}\;\times \;100\%$$

where $C_{t}$ is the drug concentration at time interval t; $C_{t-1}+C_{t-2}$ are drug concentrations prior to time interval t ($C_{0}=0)$; $V_{1}$ is the total volume of the drug release bath (25 mL), and $V_{2}$ is the volume extracted for UV-vis analysis (0.5 mL). $W_{1}$ is the initial weight of the 5-FU-loaded MSNs (0.005 g), and *L* is the drug loading capacity of the 5-FU-MSNs (taken from Equation (1)).

### 2.7. Electron Microscopy

The size and morphology of all MSNs and their drug nanoconjugates were determined by transmission electron microscopy (TEM, JEOL JEM 1010, JEOL, Tokyo, Japan) at an accelerating voltage of 100 kV and by high-resolution transmission electron microscopy (HRTEM, JEOL JEM 2100, JEOL, Tokyo, Japan) at an accelerating voltage of 100 kV. The samples for TEM and HRTEM imaging were prepared by dispersing ~5 mg MSN sample in 5 mL ethanol for 5 min in an ultra-sonic water bath. A carbon grid was then dipped into the liquid sample and allowed to dry. Spherical shaped particles were individually measured and shown in mean size distribution graphs.

The MSN surface was studied using a LEO 1450 Scanning electron Microscope (Zeiss, Oberkochen, Germany), employing SmartSEM software Version 5.03.06. The powdered samples were placed onto the front of double-sided carbon tape and affixed onto an aluminum stub. The samples were coated with gold through a BAL-TEC SCD 050 sputter coater (Leica Microsystems, Wetzlar, Germany). The scanning rate was 5 to 10 kilocounts per second using an accelerating voltage of 20 kV and a working distance of 5–10 mm.

### 2.8. Nitrogen Adsorption and Desorptio

Nitrogen adsorption and desorption isotherms of the MSNs were obtained using a Micrometrics Tri-Star II 3030 version 1.03 instrument (Micrometrics, Norcross, GA, USA) operating at 77 K. Brunauer-Emmett-Teller (BET) surface area analysis was carried out using a Micromeritics Tristar surface area and Porosity analyzer (Micrometrics, Norcross, GA, USA). The pore volume and pore diameter were calculated using the Barrett-Joyner-Halenda (BJH) method. The pore size distribution was determined using the Barrett-Joyner-Halenda (BJH) model and the desorption branch of the isotherm [30].

### 2.9. Nanoparticle Tracking Analysis (NTA)

The hydrodynamic size and zeta potential of the MSNs were analyzed using NTA (NanoSight NS500, Malvern Instruments Ltd., Worcestershire, UK). The MSN preparations contained 100 μg/mL MSN in deionized water. The particle size distribution based on the particle tracks in Brownian motion within the laser scatter volume was calculated using the Stokes-Einstein equation. Zeta potentials were calculated using the Smoluchowski approximation based on Laser-Doppler microelectrophoresis. All data collected are presented as the mode ± standard error, as calculated by NTA software v3.0.

### 2.10. Cytotoxicity

The 4,5-dimethylthiazol-2,5-diphenyltetrazolium bromide (MTT) [31] and the sulforhodamine B (SRB) assays were used to assess the cytotoxicity of the MSNs in vitro. HEK293, Caco-2, MCF-7, and HeLa cells were seeded at a density of 1 × 10^4^ cells/well in 96 well plates and were incubated at 37 °C in 5% CO_2_ for 24 h. Cells were then treated with drug loaded MSNs of different concentrations (20, 50, and 100 μg/mL) in triplicate, and incubated for 24 and 48 h. A positive control of untreated cells was included. For the MTT assay, following incubation, the medium was replaced with 200 μL fresh medium containing 20 μL of MTT solution (5 mg/ml in PBS) and incubated at 37 °C for 4 h. The MTT–medium mixture was then removed and 200 μL of DMSO added for cell permeation and solubilization of the formazan crystals, and absorbance was measured at 540 nm using a Mindray MR-96A microplate reader (Vacutec, Hamburg, Germany).

For the SRB assay, 25 μL of cold TCA (50% *w/v*) was added directly to each well and the plate incubated at 4 °C for 1 hour and then washed and air dried. Approximately 50 μL of SRB solution (0.04% *w/v*) was then added to each well and the cells incubated at room temperature for 30 min. The plate was washed four times with 200 μL of acetic acid (1% *v*/*v*) and air dried, and then 100 μL of 10 mM Tris base solution (pH 10.5) was added to each well, followed by agitation on an orbital shaker for 10 min to solubilize the protein-bound dye. The absorbance was measured as previously and growth inhibition for both assays was calculated using the following equations:

(3) $$\%\;Cell\;Growth=\;\frac{A_{540nm\;}of\;treated\;cells}{A_{540}\;controll\;cells\;\left(untreated\right)}\;\times 100\%$$

(4) % Growth Inhibition=100−% Cell Growth

### 2.11. Apoptosis

Cells were seeded into a 24-well plate at a density of 1.5 × 10^5^ cells/well and incubated for 24 h to allow for attachment. Following incubation, the medium was replaced and cells were treated with the drug nanoconjugates (50 μL/well) at pre-determined IC_50_ concentrations for 48 h, in triplicate. Untreated cells were used as the control. The medium was then removed, cells washed twice with 200 μL of PBS, and 12 μL of acridine orange:ethidium bromide dye solution (AO: EB, 1:1 *v*/*v* 1 mg/mL) was added to each well for 5 min. The excess dye was then removed, and the cells washed with 200 μL of PBS and viewed under an Olympus inverted fluorescence microscope U-RFLT50 (200× magnification) fitted with a CC12 fluorescent camera (Olympus Co., Tokyo, Japan). The apoptotic indices were calculated using the following equation:

(5) $$Apoptotic\;Index=\;\frac{Number\;of\;Apoptotic\;Cells}{Total\;Number\;of\;Counted\;Cells}\;\times 100\%$$

### 2.12. Cell Cycle Analysis

Cells were seeded and treated with the drug nanoconjugates as for the apoptosis assay. After incubation, the cells were centrifuged at 300g for 5 min. The pelleted cells were washed with PBS before resuspension in 200 μL ice cold ethanol (70% *v*/*v*). The cells were then fixed by incubation at –20 °C overnight and thereafter centrifuged and washed with PBS. Finally, 200 μL of Muse^®^ cell cycle reagent (containing propidium iodide and RNase A) was added to each tube for 30 min at room temperature in the dark. The samples were then analyzed and data generated using the Muse™ Cell Cycle software module.

### 2.13. Statistical Analyses

All data were presented as mean ± standard deviation (SD). Statistical analyses were performed using one-way analysis of variance (ANOVA) (GraphPad Prism Version 6, GraphPad Software Inc., San Diego, CA, USA). Dunnett multiple comparison and Tukey honest significant difference (HSD) tests were used as post hoc test comparatives between groups and a pre-set control, and across groups, respectively. *p* values less than 0.05 were regarded as significant. Dissolution kinetics parameters were evaluated using Microsoft Excel 2018™ and excel Add-in DD Solver software.

## 3. Results

### 3.1. Synthesis and Characterisation

**Figure 1:**
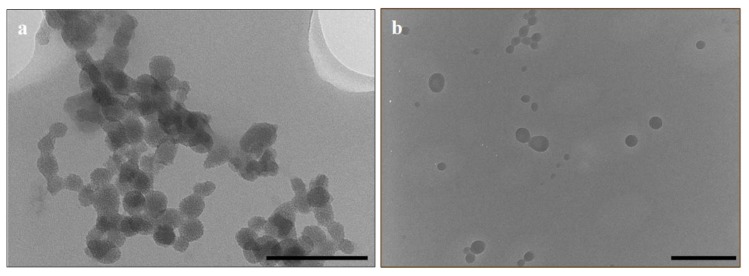
Selected transmission electron microscopy images: (**a**) Chitosan-mesoporous silica nanoparticles (CHIT-MSNs) (scale bar = 200 nm) and (**b**) 5-fluorouracil loaded 5% polyethylene glycol-CHIT-MSNs (scale bar = 500 nm).

**Figure 2:**
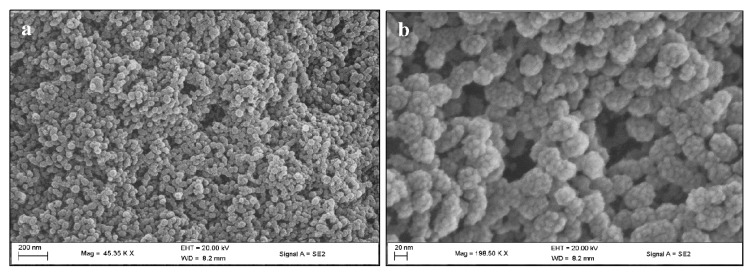
Scanning electron microscopy images of 5% PEG CHIT MSN: (**a**) scale bar = 200 nm and (**b**) scale bar = 20 nm.

Monodisperse MSNs 36 nm in size were synthesized, after which functionalization with CHIT and 2%/5% PEG increased to ~40 nm (Table 1). Upon 5-FU loading, the zeta potential of the MSN particle remained positive but was reduced, suggesting that the negatively-charged 5-FU molecules may have adhered to the free amine groups of the chitosan layer on the MSN surface. The hydrodynamic size (NTA) was slightly larger, indicating that some swelling in aqueous water may have occurred. The loading of 5-FU into the 5% PEG-CHIT-MSN formulation resulted in an almost polydisperse distribution of MSNs. This was visually assessed and confirmed by TEM and SEM images (Figure 1 and Figure 2).

The hexagonal shape of the MSNs before drug loading (Figure 2b), resulting in swelling and loss of porosity (Figure 1b), can be noted. 

**Figure 3:**
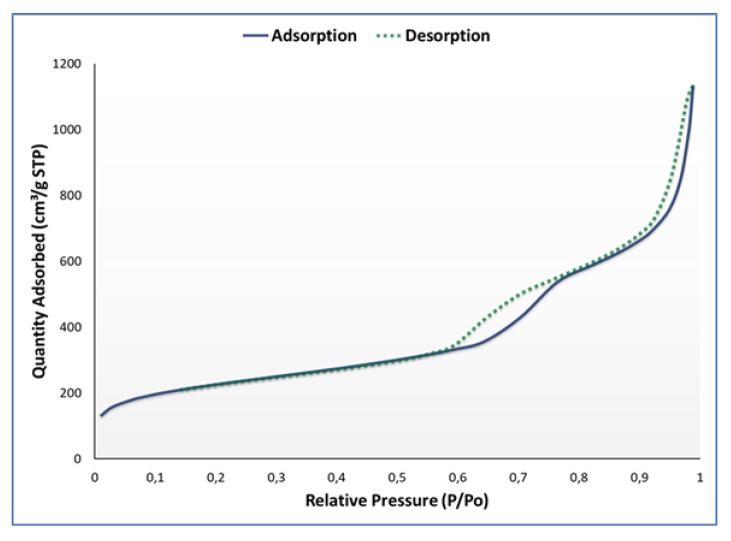
Nitrogen adsorption-desorption isotherm for synthesized MSN.

The porosity, shape, and size were evaluated using nitrogen adsorption-desorption studies prior to drug loading (Figure 3).

According to IUPAC definitions, a type IV isotherm was obtained with well-defined steps for capillary condensation and desorption in open and interstitial mesopores. Two hysteresis loops were formed at P/P_0_ = 0.6–0.75 and P/P_0_ = 0.87–0.9, which is characteristic of a mesoporous silica material with dual porosity. In accordance with the empirical classifications set out by the IUPAC, the hysteresis loops display a typical H1/H3 hybrid shape, suggesting a cylindrical/in-bottle pore shape. The shape may be attributed more to a H1 convention where the MSNs have agglomerated, with swelling of the non-rigid pores accounting for the low-pressure hysteresis pattern. Thus, the pores were defined as cylindrical, with the specific surface area and pore volume defined as 710.36 m^2^/g and 1.74 cm^2^/g. A summary of the adsorption-desorption data is provided in Table 2.

### 3.2. Drug Loading Efficiency

The loading of 5-FU relies on the physio-adsorption of the drug to the mesopores of MSN as well as the electrostatic interactions between the positively charged moieties of the polymers. The loading capacities (Table 3) shows a moderately high loading of low weighted 5-FU. Since the population was polydisperse, the loading of 5-FU into each MSN was individually regulated by the stability of the MSN and its favorable interaction with the saturated drug solution.

### 3.3. 5-FU Release and Kinetics

**Figure 4:**
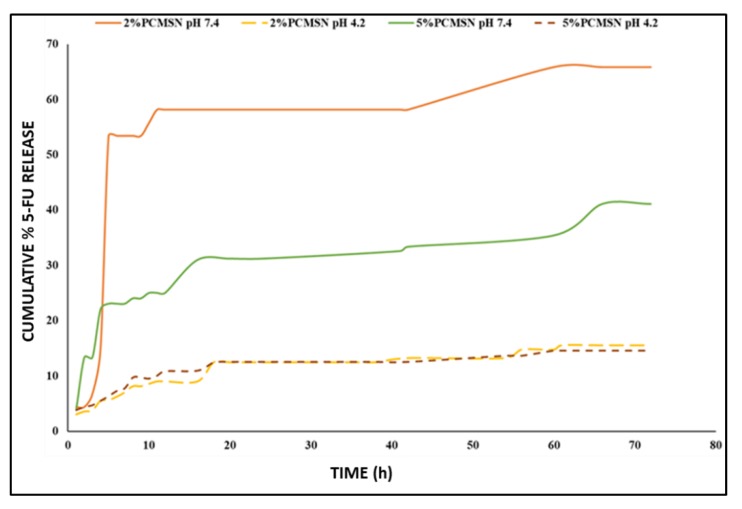
Drug release profile of 5-FU at pH 7.4 (solid lines) and pH 4.2 (long-dashed lines) for 2% PEG-CHIT-MSN (orange and yellow) and 5% PEG-CHIT-MSN (green and dark brown).

The maximum accumulated release at pH 7.4 was much higher than that at pH 4.2 (Figure 4). The release profile at pH 7.4 displayed an initial rapid release with 80% of the total drug released before 20 h. This may be equated to the rapid diffusion of 5-FU molecules that were adsorbed to the polymeric surface and at the pore entrances of the MSNs. Furthermore, at pH 7.4 there was a higher amount of positively charged species in equilibrium with negatively charged species, and as the positively charged species interacted with the PEG outer coating, more 5-FU were released.

Considering all the release profiles, the concentration gradient between the PBS buffer and the dry center of the MSN may have caused rapid diffusion of the drug into the bath medium. This was followed by slow release over a 72-hour period. The conventional drug release kinetic models tested were zero order (equation 6), first order (equation 7) [32], Higuchi (equation 8) [33], Hixson-Crowell (equation 9) [34], and Korsmeyer-Peppas (equation 10) [35]. The contribution of diffusion and erosion to the release patterns seen was evaluated and quantified using the Kopcha model (equation 11) [36]. In this model, the constants A, representative of diffusion, and B, representative of erosion, were used to mathematically illustrate which of these two factors affected the release more. According to the literature, when A/B = 1, diffusion and erosion are equal. However, when A/B < 1, erosion dominates over diffusion, and conversely for A/B > 1, the diffusion is not affected by erosion. The best release model was selected based on the correlation coefficient (R^2^) obtained, and release exponents that described the release patterns observed were defined based on the following equations:

Zero order model [37]:

(6) $$M_{t}=\;M_{0}+\;k_{0}t$$

First order model [38]:

(7) $$logM_{t}=\;loglog\;M_{0}\;+\;\frac{k_{1}t\;}{2.303}$$

Higuchi model [33]: This model assumes release from an insoluble matrix as a time-dependent progression in which Fickian diffusion is supposed.

(8) $$M_{t}=\;k_{H}\sqrt{t}$$

Hixson-Crowell model [34]: This cube root model describes release by dissolution and accounts for changes in the surface area and diameter of the particle.

(9) $${\left(M_{t}-\;M_{\infty }\right)}^{1/3}=\;k_{HC}\cdot \;t$$

Korsmeyer-Peppas model [35,39]: This model follows release from a spherical polymeric system in which there may be diffusion or erosion.

(10) $$\frac{M_{t}}{M_{\infty }}=\;k_{KP}\;.\;t^{n}$$

Kopcha model [40]: This model is used to define the amount of diffusion and erosion and their effects on the release rate.

(11) $$M_{t}=\;A\cdot \sqrt{t}+Bt$$

In these equations, $M_{0}$, $M_{t}$ and $M_{\infty }$ represent the amount of drug dissolved at time zero, time *t*, and at infinite time, respectively. The kinetic constants are represented by k and subscripted with their model initial.

The release exponent, n, is derived from the Korsmeyer–Peppas model and was used to define the release mechanism. When the *n*-value = 1, the release is zero order; if *n* = 0.43 the release is best described as Fickian diffusion where there is no relevant deformation or stresses during drug release. When 0.43 < *n* < 0.85, the release is through anomalous diffusion where there may be swelling or stress during drug release, and these structural changes may be due to temperature, activity, or structural-dimension-related fluctuations. If *n* > 0.85 there is Case II transport.

The kinetic modelling of the 5-FU release provides a detailed description of the mechanism of release and integrity of the matrix during drug delivery in vitro. The release kinetics of 5-FU at pH 7.4 could not be linearized. However, for the purposes of comparison, they are still listed in Table 4, Table 5 and Table 6.

The drug release kinetics for both PEG-CHIT-MSNs at pH 4.2, fitted into Higuchi’s mode, indicating that release occurred by diffusion. According to the Korsmeyer-Peppas fitting, the release profiles followed quasi-Fickian diffusion, and, correspondingly, a Kopcha’s model fitting displayed high A/B values and small B values, indicating release mechanisms were predominately diffusion based, with little erosion.

### 3.4. Cytotoxicity in Vitro

**Figure 5:**
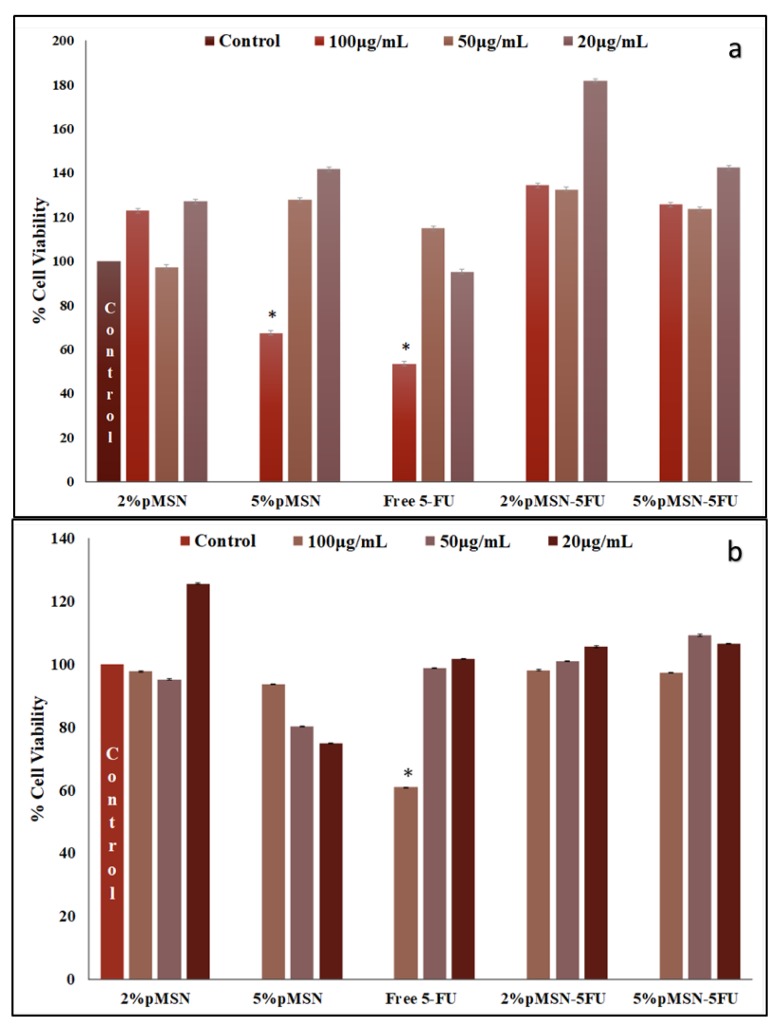
(**a**) 4,5-dimethylthiazol-2,5-diphenyltetrazolium bromide (MTT) and (**b**) sulforhodamine B (SRB) cell viability of f-MSNs and 5-FU-loaded MSNs administered at various concentrations (20, 50, and 100 μg/mL) in HEK293 cells. Data is represented as mean ± SD (*n* = 3). * *p <* 0.05 was considered statistically significant.

**Figure 6:**
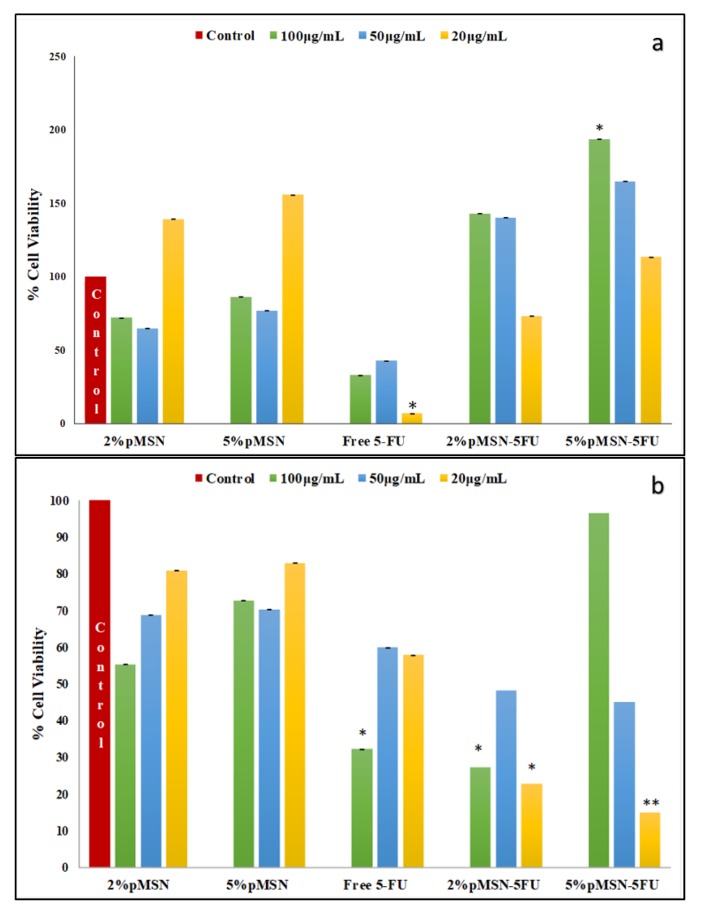
(**a**) MTT and (**b**) SRB cytotoxicity assay of f-MSNs and 5-FU-loaded MSNs administered at various concentrations (20, 50, and 100 μg/ mL) in Caco-2 cells. Data is represented as mean ± SD (*n* = 3). * *p <* 0.05 and ** *p <* 0.01 were considered statistically significant.

**Figure 7:**
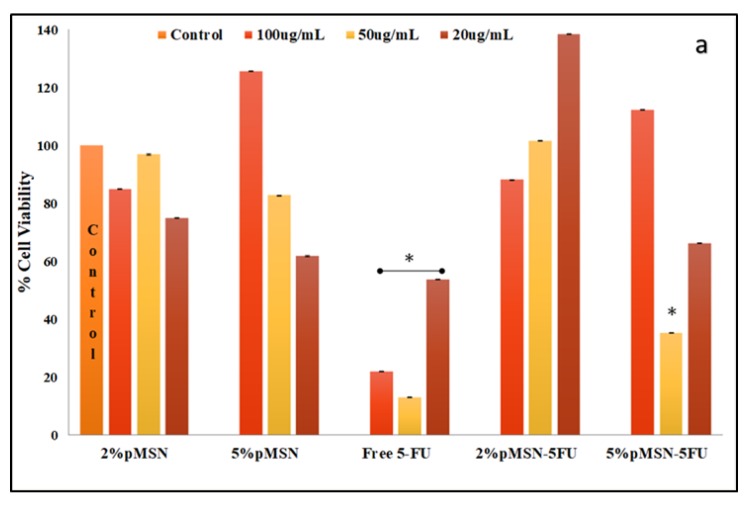

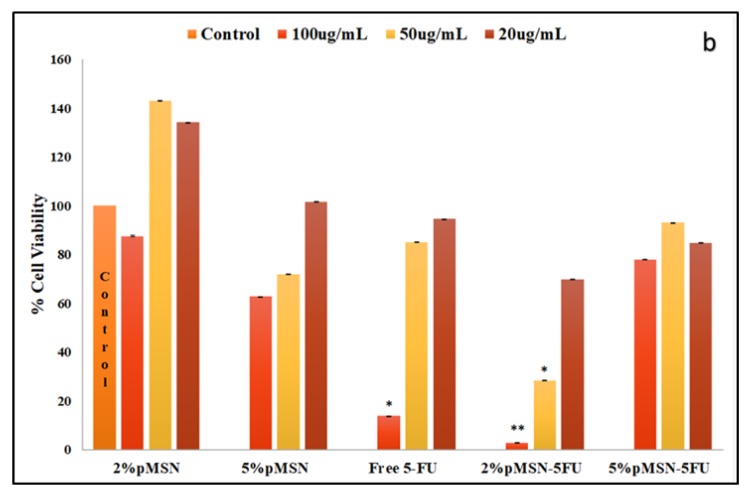
(**a**) MTT and (**b**) SRB assay of f-MSNs and 5-FU-loaded MSNs administered at various concentrations (20, 50, and 100 μg/ mL) in MCF-7 cells. Data is represented as mean ± SD (*n* = 3). * *p <* 0.05 and ** *p <* 0.01 were considered statistically significant.

**Figure 8:**
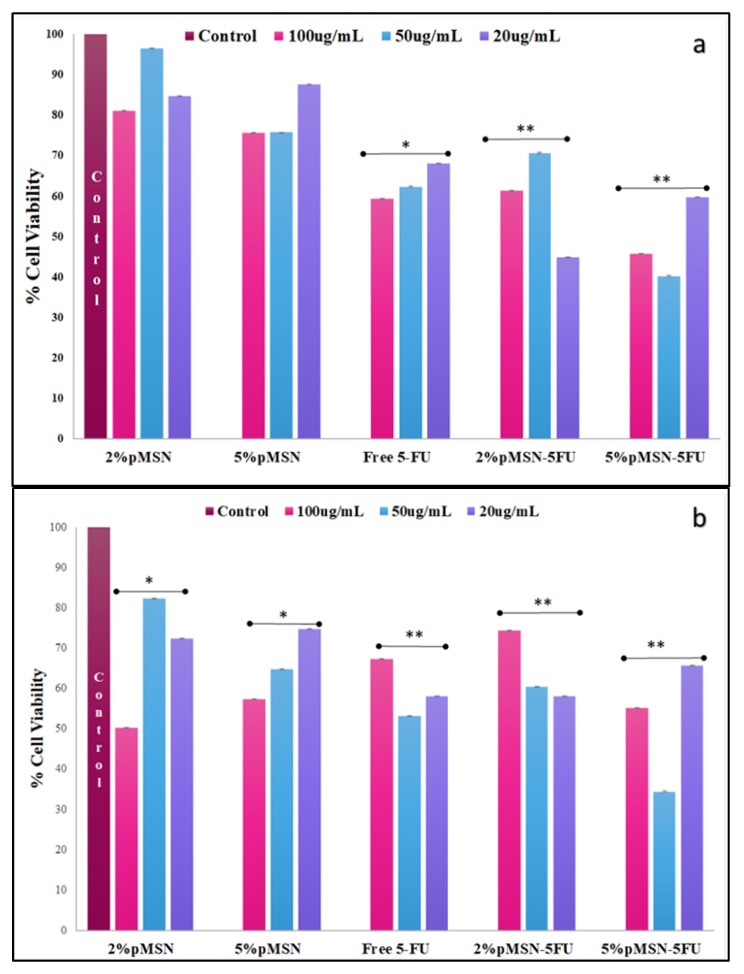
(**a**) MTT and (**b**) SRB cytotoxicity assay of f-MSNs and 5-FU-loaded MSNs administered at various concentrations (20, 50, and 100 μg/ mL) in HeLa cells. Data is represented as mean ± SD (*n* = 3). * *p <* 0.05 and ** *p <* 0.01 were considered statistically significant.

The cytotoxicity in the HEK293 cell was almost negligible, suggesting that the MSN nanocomplexes were well tolerated in this cell line (Figure 5). The Caco-2 cell viability decreased with the smallest dosage of both polymeric coated MSNs after a 48-hour duration (Figure 6). In the MCF-7 and HeLa cell lines, a larger dose of 5-FU loaded MSN was needed to elicit a significant response after the 48-hour treatment period (Figure 7 and Figure 8). The minimal concentration (Table 7) needed to inhibit 50% of the cell proliferation was then applied to further testing to elucidate and confirm the mechanism of cell death.

### 3.5. Apoptosis

**Figure 9:**
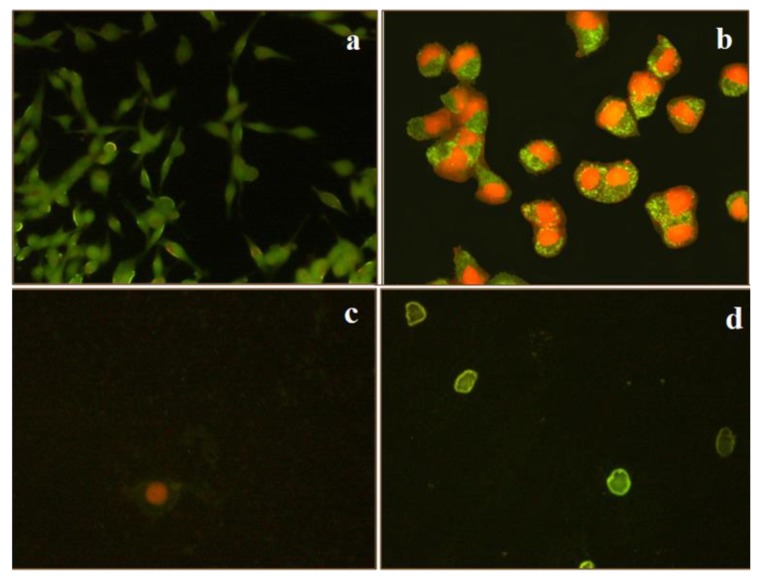
Fluorescent micrographs of dual acridine orange/ethidium bromide-stained cells showing induced morphological changes in (**a**) HEK293, (**b**) Caco-2 cells treated with 5-FU loaded 2% PEG-CHIT-MSN, (**c**) MCF-7 cell treated with 5-FU loaded 2% PEG-CHIT-MSN, and (**d**) HeLa cell treated with 5% PEG-CHIT-MSN (20× magnification).

**Figure 10:**
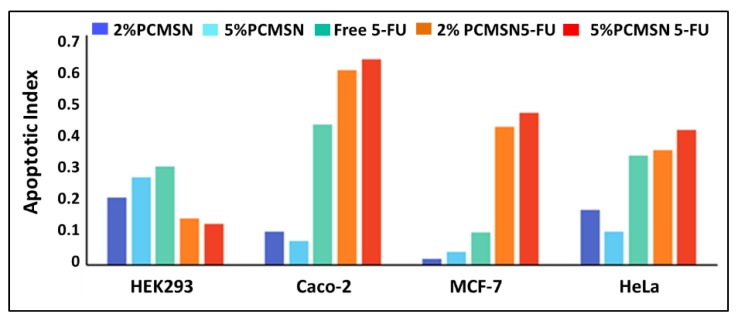
Calculated apoptotic indices for each cell line.

Characteristic apoptotic events such as membrane blebbing, formation of apoptotic bodies, chromatin condensation, and irregular cell shapes can be observed in selected fluorescent images (Figure 9). The HEK293 cells scored low apoptotic indices (Figure 10), with cells appearing morphologically unchanged and emitting a green fluorescence with all treatments (Figure 9a). Caco-2 cells were extremely 5-FU sensitive, with most cells undergoing chromatin condensation (Figure 9b). MCF-7 cells and HeLa cells showed significant morphological changes characteristic of apoptosis (Figure 9c). Treated cells produced moderately high apoptotic indices (Figure 10), with the 5% PEG-CHIT-MSN producing more of a cytostatic effect in the cancer cells.

### 3.6. Cell Cycle Analysis

**Figure 11:**
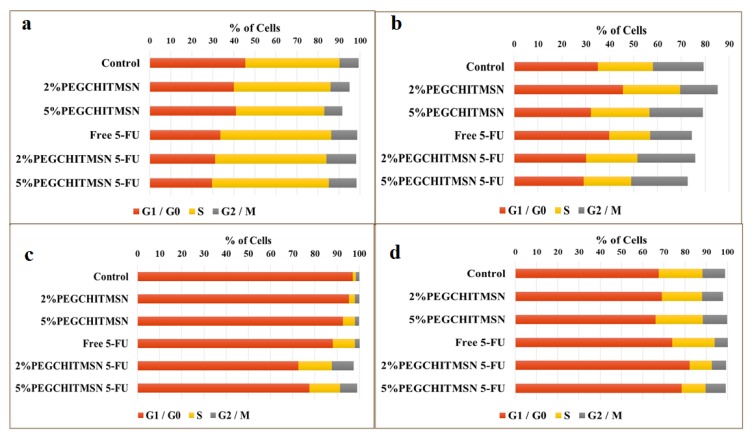
Cell cycle distribution in (**a**) HEK293, (**b**) Caco-2, (**c**) MCF-7, and (**d**) HeLa cells.

These studies corroborated results from the apoptosis assay. The HEK293 showed no significant changes in cell cycle distributions between the defined phases (Figure 11a). For the Caco-2 cells, the percentage distribution of cells between the cell cycle phases decreased and the percentage of cell debris increased, indicating that the cells had undergone apoptosis and were fragmented. Furthermore, there was a decrease in cells in the G_0_/G_1_ phase and an increase in the G_2_/M phase, which are key checkpoints for DNA damage. Thus, cells that had been arrested in G_0_/G_1_ may have selectively undergone apoptosis and cells in the G_2_/M phase may either have undergone repair mechanisms or mitotic catastrophes. Coupled with the AO/EB images (Figure 9b), it seems that the cells were more likely to have undergone mitotic catastrophe. The apoptosis results for the MCF-7 and HeLa cells were also confirmed to have undergone a slight shift in normal cell phase distributions (Figure 11). HeLa cells increased in the G_0_/G_1_ phase while MCF-7 cells saw a shift of cells in the S and G_1_/M phases (Figure 11). This was possibly due to early arrest of the S phase, as 5-FU mitigates its effect on the key replicative enzyme, thymidylate synthase.

## 4. Discussion

5-FU is a widely used chemotropic drug with a variety of commercial forms available, including oral [37,41], topical [42] and intravenous administration [6], and is often used in combination with other scheduled chemotropic drugs [43]. The wide array of toxicities experienced by patients is dependent on the metabolism of this pro-drug, as well as the individual patient’s unique epigenetic and genetic profile, coupled with their physical traits and external environmental stimuli [44,45,46,47,48,49]. Thus, the ultimate pharmacokinetic fate of 5-FU cannot be predicted prior to treatment. Hence, as in most chemotherapeutic therapies, strategies involve a trial and error base, in which patients undergo prolonged administration of repetitive dosages of potent anti-neoplastic drugs [50].

Hence, there is a need for improved delivery strategies and more biocompatible “packaging” of potent cytotoxic drugs, with a variety of engineered materials currently being evaluated for clinical and commercial applications as drug delivery vehicles [51,52,53]. MSNs are one such delivery vehicle, with extremely malleable properties that can be selectively optimized for a defined purpose.

Through a well-defined and basic sol-gel reaction, MSNs with a rigid porous framework were selectively modified with the post-synthetic addition of complimentary polymers for the enhanced and safe delivery of entrapped 5-FU. MSNs coated with a polyelectrolyte layer [20,54,55,56] have been described in the literature as a selective coating for many recent drug delivery vehicles, as they allow for selective controlled-release properties.

A uniform population of spherical MSNs ranging between 36–65 nm, with a large surface area averaging 710.3616 m^2^/g and cylindrical pores averaging 7.4 nm with a pore volume of approximately 1.743321 cm^3^/g, were incompletely capped with the addition of an interfacial layer of CHT and PEG moieties. The functionalized superficial area resulted in an increase in hydrophilicity, consequently eliciting a pH-sensitive gating of entrapped 5-FU molecules. Importantly, the polymerization of the porous framework, encapsulating the chemotropic 5-FU, demonstrated biostability and biocompatibility, and decreased the cytotoxicity of 5-FU encapsulated MSN formulations in vitro. This is supported in the literature, with silica material exhibiting low toxicity and an overall safe pharmacokinetic fate leading to the use of silica as an FDA-approved material for biological systems [50,57]

The loading of 5-FU was moderate, with this low molecular weight (130.08 g/mol) particle most likely adsorbing to the positively-charged amine groups of CHIT, which were ultimately covered by a brush-like layer of PEG which may have reduced the extensive intake of 5-FU molecules. This may have entrapped 5-FU in both the polymer-capped superficial layer and interior pores of MSN. Thus, when immersed in solution, 5-FU was rapidly released from the polymerized superficial layer, followed by a slow and gradual release from the MSN matrix over the 72-h period. Hydrophilic-related shrinking of the 5-FU-MSN formulations may have hindered entrapped drug that was too tightly compacted in the matrix. At a pH of 7.4, the maximal 5-FU drug release after 72 hours was recorded as 66% for the 2% PEG-CHIT-MSN and 41% for the 5% PEG-CHIT-MSN. At pH 4.2 the maximum 5-FU release was recorded as 16% and 15% for the 2% PEG-CHIT-MSN and 5%PEG-CHIT-MSN, respectively. Thus, the in vitro release of 5-FU from the designed polymeric MSN reached a maximal concentration of 0.01 mg/mL (per 0.2 mg/mL MSN:PBS) at pH 7.4. The 2% PEG-CHIT-MSN released the most 5-FU at pH 7.4 while both the 2% and 5% PEG-CHIT-MSNs released similar total percentages of 5-FU at pH 4.2, indicating shared kinetic mechanisms influencing release patterns in acidic conditions.

The release kinetics at pH 7.4 were non-linear, suggesting release was subject to saturation of one of the pharmacokinetic measures [58]. The models utilized in this study only accounted for the rate of diffusion and whether there was erosion or diffusion. However, a more suitable model could be a multi-faceted one that includes the effects of solute diffusion co-efficients, electrostatic interaction between 5-FU and the polymer/drug matrix, and the heterogenous structure of drug delivery systems [58,59,60]. The synthesized MSNs, especially the 5% PEG-CHIT-MSN, displayed polydispersity while presenting a heterogeneously sized population. Thus, the release mechanism could not be linearized for the heterogenous distributed samples, especially at pH 7.4, where there was increased electrostatic interaction between the polymer surroundings and the 5-FU encased in the MSN [61,62]. The release of 5-FU may thus be subject to pH-gradients, with burst-release occurring at a more neutral pH than under acidic pH conditions [63].

Burst release kinetics would be associated with the MSN’s geometry, surface characteristics, the heterogenous distribution of the drug in the MSN matrix, the intrinsic dissolution rate of 5-FU, the innate heterogeneity of the porous matrix, and the pore densities [57,58,63]. Thus, a more conclusive model that allows for the assessment of burst release together with slow-controlled release would be ideal to elucidate the behavior of these drug-loaded MSNs [59].

In this work, the cytotoxic, apoptotic, and cell cycle activities of the MSNs were evaluated in four human cancer cell lines to assess the biological performance of MSNs as a drug delivery vehicle in relation to its investigated kinetic release profiles. Supporting previous literature relating the relative biocompatibility and safe pharmacokinetic fate of MSNs, this study found that spherical, weakly positively charged MSNs which were polymer-coated with CHIT and PEG moieties and loaded with 5-FU showed favorable uptake in cancer cell lines, with potent induced cytotoxic, apoptotic and cell-cycle distribution shift events being derived from 48-h exposure treatments in colon, breast, and cervical cancer cells undergoing rapid apoptotic events and shifts in cell cycle distribution. Both apoptotic analyses and cell cycle distribution shifts suggested treatment-dependent induction of cell cycle arrest, as 5-FU mitigated its effects as a synthase inhibitor. Additionally, cytotoxicity results supported an effective reduction of the cancerous cell population at therapeutically relevant dosages. Importantly, no critical cytotoxicity, apoptotic events, or cell cycle shifts occurred within the HEK293 model, alluding to a decreased 5-FU-MSN conjugate uptake into healthy, dividing cells [57,64,65,66].

Overall, the favorable combination of CHIT and PEG as a polyelectrolyte coating on synthesized MSNs bestowed beneficial properties upon loaded 5-FU molecules in vitro, suggesting the possible reduction of commonly-seen chemotherapeutic-induced side effects in healthy tissue at a lower dosage and exposure period required to necessitate a response. Thus, there is therapeutic relevancy attached to the use of polymeric coated MSNs loaded with 5-FU for drug delivery in cancer therapy.

## 5. Conclusions

The selective and exaggerated burst-release until saturation is reached alludes to the potential use of these MSN loaded 5-FU formulations in orally administered regimens for colorectal cancers or cancers of the gastrointestinal tract, as the small intestine has a more basic pH, which favors 5-FU release from MSN without triggering systemic toxicity in healthy tissue or accumulation in non-targeted organs. Furthermore, MSN is a relatively biodegradable material that has been commercially and clinically applied as additives in a variety of products with no adverse toxicities noted. Thus, an oral administration of 5-FU would be highly advantageous, as the matrix is capable of sustained drug release through Fickian diffusion with little erosion or degradation of the polymeric framework in vitro. Pharmacokinetic studies have further alluded to the safe excretion of silica from the body once metabolized. Hence, further studies and in vivo investigations are warranted to enable a transition to possible clinical applications.

## Figures and Tables

**Table 1:** Transmission electron microscopy (TEM) size, polydispersity index (PDI), hydrodynamic size, and zeta potential of all mesoporous silica nanoparticles (MSNs) and 5-fluorouracil-loaded mesoporous silica nanoparticles.

Nanoparticle	Mean Diameter (TEM) (nm ± Standard Deviation)	PDI (SD/mean)^2^	Hydrodynamic Size (Nanoparticle Tracking Analysis) (nm ± SD)	Zeta Potential (mV)
MSN	36.09 ± 7.08	0.0385	188 ± 51.6	−9.8 ± 1
CHITOSAN (CHIT)-MSN	39.43 ± 7.22	0.0335	62.2 ± 16	32.4 ± 0.4
2% Polyethylene glycol (PEG)-CHIT-MSN	40.75 ± 7.11	0.0422	12 ± 3.3	17.0 ± 16.5
5% PEG-CHIT-MSN	40.37 ± 7.70	0.0364	54.8 ± 2.1	7.4 ± 0.7
2% PEG-CHIT-MSN-5FU	48.32 ± 8.20	0.0287	54.8 ± 40.7	11.2 ± 7.0
5% PEG-CHIT-MSN-5FU	64.54 ± 25.11	0.1514	47.4 ± 5.5	3.4 ± 0.0

**Table 2:** Summary of nitrogen adsorption-desorption data for synthesized MSNs.

**Surface Area**	
Brunauer-Emmet-Teller (BET) surface area	809.4447 m^2^/g
t-Plot micropore area	162.2294 m^2^/g
t-Plot external surface area	647.2153 m^2^/g
Barrett-Joyner-Halenda (BJH) adsorption cumulative surface area of pores between 17.000 Å and 3000.000 Å diameter	680.013 m^2^/g
BJH desorption cumulative surface area of pores between 17.000 Å and 3000.000 Å diameter	710.3616 m^2^/g
**Pore Volume**	
Single point adsorption total pore volume of pores less than 1047.206 Å diameter at P/Po = 0.981156675	1.529764 cm^3^/g
t-Plot micropore volume	0.066025 cm^3^/g
BJH adsorption cumulative volume of pores between 17.000 Å and 3000.000 Å diameter	1.726356 cm^3^/g
BJH desorption cumulative volume of pores between 17.000 Å and 3000.000 Å diameter	1.743321 cm^3^/g
**Pore Size**	
Adsorption average pore width (4V/A by BET)	75.5957 Å
BJH adsorption average pore diameter (4V/A)	101.548 Å
BJH desorption average pore diameter (4V/A)	98.165 Å
Average particle size	74.125 Å

**Table 3:** Loading capacity of polymeric MSNs with 5-FU.

5-FU Loaded MSNs		
	5% PEG-CHIT-MSN	2% PEG-CHIT-MSN
Loading capacity (%)	18.02	15.02
Loading capacity (mg 5-FU/mg f-MSN)	0.1802	0.1502

**Table 4:** Correlation co-efficient (*R*^2^) obtained from modelling 5-FU-loaded 2% PEG-CHIT-MSNs through release kinetic models at pH 7.4 and 4.2.

pH	Zero-Order	First-Order	Higuchi’s	Hixson-Crowell’s	Korsmeyer-Peppas’	Kopcha’s
Correlation value (*R*^2^)						
4.2	0.81	0.55	0.91	0.27	0.95	0.98
7.4	0.35	0.26	0.48	0.64	0.65	0.16

**Table 5:** Correlation co-efficient (*R*^2^) obtained from modelling 5-FU loaded 5% PEG-CHIT-MSNs through release kinetic models at pH 7.4 and 4.2.

pH	Zero-Order	First-Order	Higuchi’s	Hixson-Crowell’s	Korsmeyer-Peppas’	Kopcha’s
Correlation value (*R*^2^)						
4.2	0.72	0.44	0.85	0.72	0.91	0.97
7.4	0.69	0.29	0.81	0.51	0.75	0.70

**Table 6:** Korsmeyer-Peppas model’s release exponent factor and the corresponding Kopcha’s release model fitting results.

**pH 4.2**				
**Multidrug Formulation**	**Korsmeyer-Peppas Model**	**Kopcha Model**		
	**n-value**	**A**	**B**	**A/B**
2% PEG-CHIT-MSN	0.39	2.97	0.13	22.85
5% PEG-CHIT-MSN	0.32	3.76	0.27	13.93
**pH 7.4**				
**Multidrug Formulation**	**Korsmeyer-Peppas Model**	**Kopcha Model**		
	**n-value**	**A**	**B**	**A/B**
2% PEG-CHIT-MSN	0.74	5.16	2.02	2.55
5% PEG-CHIT-MSN	0.35	6.87	0.16	42.94

**Table 7:** IC_50_ values of 5-FU-MSN treated tested cell lines (Figure 4, Figure 5, Figure 6 and Figure 7).

	2% PEG-CHIT-MSN 5-FU	5% PEG-CHIT-MSN 5-FU
**HEK293**	-	-
**MCF-7**	91 μg/mL (48 h)	168 μg/mL (48 h)
**Caco-2**	84 μg/mL (48 h)	109 μg/mL (48 h)
**HeLa**	85 μg/mL (48 h)	54 μg/mL (48 h)

## References

[1] Diasio R.B., Harris B.E. (1989). Clinical Pharmacology of 5-Fluorouracil. Clin. Pharmacokinet..

[2] Zoli W., Ulivi P., Tesei A., Fabbri F., Rosetti M., Maltoni R., Giunchi D.C., Ricotti L., Brigliadori G., Vannini I. (2005). Addition of 5-fluorouracil to doxorubicin-paclitaxel sequence increases caspase-dependent apoptosis in breast cancer cell lines. Breast Cancer Res..

[3] Groves T.R., Farris R., Anderson J.E., Alexander T.C., Kiffer F., Carter G., Wang J., Boerma M., Allen A.R. (2017). 5-Fluorouracil chemotherapy upregulates cytokines and alters hippocampal dendritic complexity in aged mice. Behav. Brain Res..

[4] Akinyelu J., Singh M. (2019). Folate-tagged chitosan functionalized gold nanoparticles for enhanced delivery of 5-fluorouracil to cancer cells. Appl. Nanosci..

[5] Yoshikawa R., Kusunoki M., Yanagi H., Noda M., Furuyama J.I., Yamamura T., Hashimoto-Tamaoki T. (2001). Dual antitumor effects of 5-fluorouracil on the cell cycle in colorectal carcinoma cells: A novel target mechanism concept for pharmacokinetic modulating chemotherapy. Cancer Res..

[6] Martin M., Villar A., Sole-Calvo A., Gonzalez R., Massuti B., Lizon J., Camps C., Carrato A., Casado A., Candel M.T. (2003). Doxorubicin in combination with fluorouracil and cyclophosphamide (i.v. FAC regimen, day 1, 21) versus methotrexate in combination with fluorouracil and cyclophosphamide (i.v. CMF regimen, day 1, 21) as adjuvant chemotherapy for operable breast cancer: A study by the GEICAM group. Ann. Oncol. Off. J. Eur. Soc. Med. Oncol..

[7] Lopez M., Papaldo P., Di Lauro L., Vici P., Carpano S., Conti E.M.S. (1989). 5-Fluorouracil, Adriamycin, Cyclophosphamide (FAC) vs. 5-Fluorouracil, Epirubicin, Cyclophosphamide (FEC) in Metastatic Breast Cancer. Oncology.

[8] Van Kuilenburg A.B.P. (2004). Dihydropyrimidine dehydrogenase and the efficacy and toxicity of 5-fluorouracil. Eur. J. Cancer.

[9] Wang A.Z., Langer R., Farokhzad O.C. (2012). Nanoparticle Delivery of Cancer Drugs. Annu. Rev. Med..

[10] Anselmo A.C., Mitragotri S. (2015). A Review of Clinical Translation of Inorganic Nanoparticles. AAPS J..

[11] Jahangirian H., Lemraski E.G., Webster T.J., Rafiee-Moghaddam R., Abdollahi Y. (2017). A review of drug delivery systems based on nanotechnology and green chemistry: Green nanomedicine. Int. J. Nanomed..

[12] Tang F., Li L., Chen D. (2012). Mesoporous silica nanoparticles: Synthesis, biocompatibility and drug delivery. Adv. Mater..

[13] Kwon S., Singh R.K., Perez R.A., Abou Neel E.A., Kim H.-W., Chrzanowski W. (2013). Silica-based mesoporous nanoparticles for controlled drug delivery. J. Tissue Eng..

[14] Wang Y., Zhao Q., Han N., Bai L., Li J., Liu J., Che E., Hu L., Zhang Q., Jiang T. (2015). Mesoporous silica nanoparticles in drug delivery and biomedical applications. Nanomed. Nanotechnol. Biol. Med..

[15] Park J.H., Ye M., Park K. (2005). Biodegradable polymers for microencapsulation of drugs. Molecules.

[16] Barrett W.E., Bianchine J.R. (1975). The bioavailability of ultramicrosize griseofulvin (Gris-PEG) tablets in man. Curr. Ther. Res. Clin. Exp..

[17] Kumari A., Yadav S.K., Yadav S.C. (2010). Biodegradable polymeric nanoparticles based drug delivery systems. Coll. Surf. B Biointerf..

[18] Näkki S., Rytkönen J., Nissinen T., Florea C., Riikonen J., Ek P., Zhang H., Santos H.A., Närvänen A., Xu W. (2015). Improved stability and biocompatibility of nanostructured silicon drug carrier for intravenous administration. Acta Biomater..

[19] Verhoef J.J.F., Anchordoquy T.J. (2013). Questioning the Use of PEGylation for Drug Delivery. Drug. Deliv. Transl. Res..

[20] Feng W., Zhou X., He C., Qiu K., Nie W., Chen L., Wang H., Mo X., Zhang Y. (2013). Polyelectrolyte multilayer functionalized mesoporous silica nanoparticles for pH-responsive drug delivery: Layer thickness-dependent release profiles and biocompatibility. J. Mater. Chem. B.

[21] Zhao Y., Sun X., Zhang G., Trewyn B.G., Slowing I.I., Lin V.S.-Y. (2011). Interaction of Mesoporous Silica Nanoparticles with Human Red Blood Cell Membranes: Size and Surface Effects. ACS Nano.

[22] Gref R., Lück M., Quellec P., Marchand M., Dellacherie E., Harnisch S., Blunk T., Müller R.H. (2000). “Stealth” corona-core nanoparticles surface modified by polyethylene glycol (PEG): Influences of the corona (PEG chain length and surface density) and of the core composition on phagocytic uptake and plasma protein adsorption. Coll. Surf. B. Biointerf..

[23] Yin Win K., Feng S.-S. (2005). Effects of particle size and surface coating the cellular uptake of polymeric nanoparticles. Biomaterials.

[24] Lu F., Wu S.-H., Hung Y., Mou C.-Y. (2009). Size Effect on Cell Uptake in Well-Suspended, Uniform Mesoporous Silica Nanoparticles. Small.

[25] Tourne-Peteilh C., Bégu S., Lerner D., Galarneau A., Lafont U., Devoisselle J.-M. (2012). Sol–gel one-pot synthesis in soft conditions of mesoporous silica materials ready for drug delivery system. J. Sol-Gel Sci. Technol..

[26] Vazquez N.I., Gonzalez Z., Ferrari B., Castro Y. (2017). Synthesis of mesoporous silica nanoparticles by sol–gel as nanocontainer for future drug delivery applications. Boletín la Soc. Española Cerámica y Vidr..

[27] Hu Y., Ke L., Chen H., Zhuo M., Yang X., Zhao D., Zeng S., Xiao X. (2017). Natural material-decorated mesoporous silica nanoparticle container for multifunctional membrane-controlled targeted drug delivery. Int. J. Nanomed..

[28] Wang J., Liu H., Leng F., Zheng L., Yang J., Wang W., Huang C.Z. (2014). Autofluorescent and pH-responsive mesoporous silica for cancer-targeted and controlled drug release. Microporous Mesoporous Mater..

[29] She X., Chen L., Velleman L., Li C., Zhu H., He C., Wang T., Shigdar S., Duan W., Kong L. (2015). Fabrication of high specificity hollow mesoporous silica nanoparticles assisted by Eudragit for targeted drug delivery. J. Coll. Interf. Sci..

[30] Barrett E.P., Joyner L.G., Halenda P. (1951). The determination of pore volume and area distribution in porous substances. Vol. Area Distrib. Porous Subst..

[31] Mosmann T. (1983). Rapid colorimetric assay for cellular growth and survival: Application to proliferation and cytotoxicity assays. J. Immunol. Methods.

[32] Dash S., Murthy P., Nath L., Chowdhury P. (2010). Kinetic modeling on drug release from controlled drug delivery systems. Acta Pol. Pharm..

[33] Higuchi T. (1963). Mechanism of sustained-action medication. Theoretical analysis of rate of release of solid drugs dispersed in solid matrices. J. Pharm. Sci..

[34] Hixson A.W., Crowell J.H. (1931). Dependence of Reaction Velocity upon surface and Agitation. Ind. Eng. Chem..

[35] Korsmeyer R.W., Lustig S.R., Peppas N.A. (1986). Solute and penetrant diffusion in swellable polymers. I. Mathematical modeling. J. Polym. Sci. Part B Polym. Phys..

[36] Kopcha M., Tojo K., Lordi N.G. (1990). Evaluation of methodology for assessing release characteristics of thermosoftening vehicles. J. Pharm. Pharmacol..

[37] Sankalia J.M., Sankalia M.G., Mashru R.C. (2008). Drug release and swelling kinetics of directly compressed glipizide sustained-release matrices: Establishment of level A IVIVC. J. Control. Rel..

[38] Mulye N.V., Turco S.J. (1995). A Simple Model Based on First Order Kinetics to Explain Release of Highly Water Soluble Drugs from Porous Dicalcium Phosphate Dihydrate Matrices. Drug Dev. Ind. Pharm..

[39] Korsmeyer R.W., Gurny R., Doelker E., Buri P., Peppas N.A. (1983). Mechanisms of solute release from porous hydrophilic polymers. Int. J. Pharm..

[40] Kopcha M., Lordi N.G., Tojo K.J. (1991). Evaluation of Release from Selected Thermosoftening Vehicles. J. Pharm. Pharmacol..

[41] Anirudhan T.S., Vasantha C.S., Sasidharan A.V. (2017). Layer-by-layer assembly of hyaluronic acid/carboxymethylchitosan polyelectrolytes on the surface of aminated mesoporous silica for the oral delivery of 5-fluorouracil. Eur. Polym. J..

[42] Werschler W.P. (2008). Considerations for use of Fluorouracil cream 0.5% for the treatment of actinic keratosis in elderly patients. J. Clin. Aesthet. Dermatol..

[43] Grem J.L. (2000). 5-Fluorouracil: Forty-Plus and Still Ticking. A Review of its Preclinical and Clinical Development. Invest. New Drugs.

[44] Montagnoli A., Valsasina B., Croci V., Menichincheri M., Rainoldi S., Marchesi V., Tibolla M., Tenca P., Brotherton D., Albanese C. (2008). A Cdc7 kinase inhibitor restricts initiation of DNA replication and has antitumor activity. Nat. Chem. Biol..

[45] Scartozzi M., Maccaroni E., Giampieri R., Pistelli M., Bittoni A., Del Prete M., Berardi R., Cascinu S. (2011). 5-fluorouracil pharmacogenomics: Still rocking after all these years?. Pharmacogenomics.

[46] Jarzembska K.N., Kubsik M., Kamiński R., Woźniak K., Dominiak P.M. (2012). From a Single Molecule to Molecular Crystal Architectures: Structural and Energetic Studies of Selected Uracil Derivatives. Cryst. Growth Des..

[47] Barnett S.A., Hulme A.T., Tocher D.A. (2006). 5-Fluorouracil and thymine form a crystalline solid solution. Acta Crystallogr. Sect. C Cryst. Struct. Commun..

[48] Hulme A.T., Price S.L., Tocher D.A. (2005). A New Polymorph of 5-Fluorouracil Found Following Computational Crystal Structure Predictions. J. Am. Chem. Soc..

[49] Fischer J., Ganellin C.R. (2006). Analogue-Based Drug Discovery.

[50] Pullarkat S.T., Stoehlmacher J., Ghaderi V., Xiong Y-P., Ingles S.A., Sherrod A., Warren R., Tsao-Wei D., Groshen S., Lenz H-J. (2001). Thymidylate synthase gene polymorphism determines response and toxicity of 5-FU chemotherapy. Pharmacogenomics J..

[51] Sarker D. (2005). Engineering of Nanoemulsions for Drug Delivery. Curr. Drug Deliv..

[52] Elsabahy M., Wooley K.L. (2012). Design of polymeric nanoparticles for biomedical delivery applications. Chem. Soc. Rev..

[53] Wang M., Thanou M. (2010). Targeting nanoparticles to cancer. Pharmacol. Res..

[54] Zhu Y., Shi J., Shen W., Dong X., Feng J., Ruan M., Li Y. (2005). Stimuli-Responsive Controlled Drug Release from a Hollow Mesoporous Silica Sphere/Polyelectrolyte Multilayer Core–Shell Structure. Angewandte Chemie..

[55] Huang Y., Yu H., Xiao C. (2007). pH-Sensitive Cationic Guar Gum/Poly (Acrylic Acid) Polyelectrolyte Hydrogels: Swelling and In Vitro Drug Release. Carbohydrate Polymers..

[56] Yang Y., Tao X., Hou Q., Ma Y., Chen X.-L., Chen J.-F. (2010). Mesoporous Silica Nanotubes Coated with Multilayered Polyelectrolytes for pH-Controlled Drug Release. Acta Biomaterialia.

[57] Kulkarni A.R., Soppimath K.S., Aminabhavi T.M., Rudzinski W.E. (2001). In-vitro release kinetics of cefadroxil-loaded sodium alginate interpenetrating network beads. Eur. J. Pharm. Biopharm..

[58] Barzegar-Jalali M., Adibkia K., Valizadeh H., Reza M., Shadbad S., Nokhodchi A., Omidi Y., Mohammadi G., Nezhadi S.H., Hasan M. (2008). Kinetic Analysis of Drug Release from Nanoparticles. J. Pharm. Pharm. Sci..

[59] Fu Y., Kao W.J. (2010). Drug release kinetics and transport mechanisms of non-degradable and degradable polymeric delivery systems. Expert Opin. Drug Deliv..

[60] Costa P., Sousa Lobo J.M. (2003). Evaluation of mathematical models describing drug release from estradiol transdermal systems. Drug Dev. Ind. Pharm..

[61] Ritger P.L., Peppas N.A. (1987). A simple equation for description of solute release II. Fickian and anomalous release from swellable devices. J. Control. Rel..

[62] Chen T., Wu W., Xiao H., Chen Y., Chen M., Li J. (2016). Intelligent Drug Delivery System Based on Mesoporous Silica Nanoparticles Coated with an Ultra-pH-Sensitive Gatekeeper and Poly(ethylene glycol). ACS Macro Lett..

[63] Gbureck U., Vorndran E., Müller F.A., Barralet J.E. (2007). Low temperature direct 3D printed bioceramics and biocomposites as drug release matrices. J. Control. Rel..

[64] Watermann A., Brieger J. (2017). Mesoporous Silica Nanoparticles as Drug Delivery Vehicles in Cancer. Nanomaterials.

[65] Kusaczuk M., Krętowski R., Naumowicz M., Stypułkowska A., Cechowska-Pasko M. (2018). Silica nanoparticle-induced oxidative stress and mitochondrial damage is followed by activation of intrinsic apoptosis pathway in glioblastoma cells. Int. J. Nanomed..

[66] Babaei M., Abnous K., Taghdisi S.M., Amel Farzad S., Peivandi M.T., Ramezani M., Alibolandi M. (2017). Synthesis of theranostic epithelial cell adhesion molecule targeted mesoporous silica nanoparticle with gold gatekeeper for hepatocellular carcinoma. Nanomedicine.

